# Primary hepatic neuroendocrine neoplasms: imaging characteristics and misdiagnosis analysis

**DOI:** 10.3389/fonc.2024.1391663

**Published:** 2024-05-14

**Authors:** Xiu-Rong Yang, Ying-Li Li, Zi-Yan Li, Xiao-Ming Chai

**Affiliations:** ^1^ Department of Radiology, Xing Lin Branch of the First Affiliated Hospital of Xiamen University, School of Medicine, Xiamen University, Xiamen, China; ^2^ Department of Radiology, The First Affiliated Hospital of Xiamen University, School of Medicine, Xiamen University, Xiamen, China; ^3^ The Third Clinical College of Fujian Medical University, Fuzhou, China

**Keywords:** diagnosis, tomography, x-ray computed, magnetic resonance imaging, neuroendocrine neoplasms, liver

## Abstract

**Objective:**

To analyze the CT and MR features of Primary hepatic neuroendocrine neoplasms (PHNENs) in order to enhance the diagnostic accuracy of this disease.

**Methods:**

A retrospective analysis was conducted on patients diagnosed with hepatic neuroendocrine neoplasms, excluding other sites of origin through general examination and postoperative follow-up. The CT and MR signs were analyzed according to the 2018 version of Liver Imaging Reporting and Data System (LI-RADS), along with causes of misdiagnosis.

**Results:**

Twelve patients, including 6 males and 6 females, were enrolled in this study. There was no significant increase in liver tumor markers among all cases. Most masses were multiple (9/12), exhibiting low attenuation on pre-contrast CT scans, T1-hypointense signal, T2-hyperintense signal, and restricted diffusion. The majority of these masses (7/10) demonstrated similar rim arterial phase hyper-enhancement as well as peripheral “washout” during venous portal phase and delayed phase imaging. Three cases had incomplete capsules while one case had a complete capsule. Cyst/necrosis was observed in 7 out of all cases following administration of contrast agent, with 5 mainly distributed in the periphery. All masses lacked fat, calcification, vascular or bile duct tumor thrombus formation.

**Conclusion:**

The imaging findings associated with PHNENs possess certain specificity, often presenting as multiple masses within the liver accompanied by peripheral cyst/necrosis, similar rim arterial phase hyper-enhancement during venous portal phase and delayed phase imaging.

## 1 Background

Neuroendocrine neoplasms (NENs) are rare tumors originating from peptidergic neurons and neuroendocrine cells, exhibiting neuroendocrine differentiation and expression of neuroendocrine markers. NENs can manifest in various locations throughout the body, with a higher prevalence observed in the lung, gastrointestinal tract, and pancreas (1). The annual incidence rate of NENs among all GI tumors is approximately 2 per 100,000 individuals. While the liver serves as the most common site for metastasis in NEN cases, Primary hepatic neuroendocrine neoplasms (PHNENs) remain exceedingly uncommon. Since Edmondson et al.’s initial case report in 1958 (2), fewer than 150 instances of PHNENs have been documented within existing literature. PHNENs accounts for merely 0.46% of all primary liver tumors and represents only 0.8%-4.0% of total neuroendocrine tumor cases (3). Previous reports primarily describe adult patients affected by PHNENs (age range: 8-83 years; Mean: 50 years), with a slightly higher occurrence rate among females (4, 5). In recent studies conducted by Song et al., out of a cohort comprising 517 NEN patients, they reported on fifteen occurrences of PHNENs accounting for approximately 2.7% of all NENS cases - consistent with earlier findings (6).

The clinical manifestations and imaging features of PHNENs lack specificity, often leading to misdiagnosis as hepatocellular carcinoma, cholangiocarcinoma, hepatic adenoma, hemangioma, among others. Currently, there are no established guidelines for the treatment of PHNENs. Individualized treatment plans should consider tumor location, stage and differentiation degree, patient age, comorbidities and symptoms while adopting a multidisciplinary approach. Viable therapeutic options include surgical intervention, chemotherapy administration, radiotherapy treatment, transcatheter arterial chemoembolization procedure and utilization of somatostatin analogs (7–10). According to the expert consensus on metastatic hepatic neuroendocrine neoplasms (NENs) (11), surgical resection remains advantageous for the majority of patients. Therefore, early diagnosis holds paramount importance in treatment decision-making and favorable prognosis for patients with PHNENs. The imaging manifestations of PHNENs reported in previous literature mainly consist of case reports (12, 13) and lack systematic analysis. Hence, this study retrospectively analyzed clinical, pathological, and imaging data from 12 patients with PHNENs to enhance the accuracy of preoperative diagnosis.

## 2 Materials and methods

### 2.1 Experimental design

This study received approval from the ethics committee of our hospital ([2024] NO. 005), and informed consent was waived for the subjects.”

**Figure 1:**
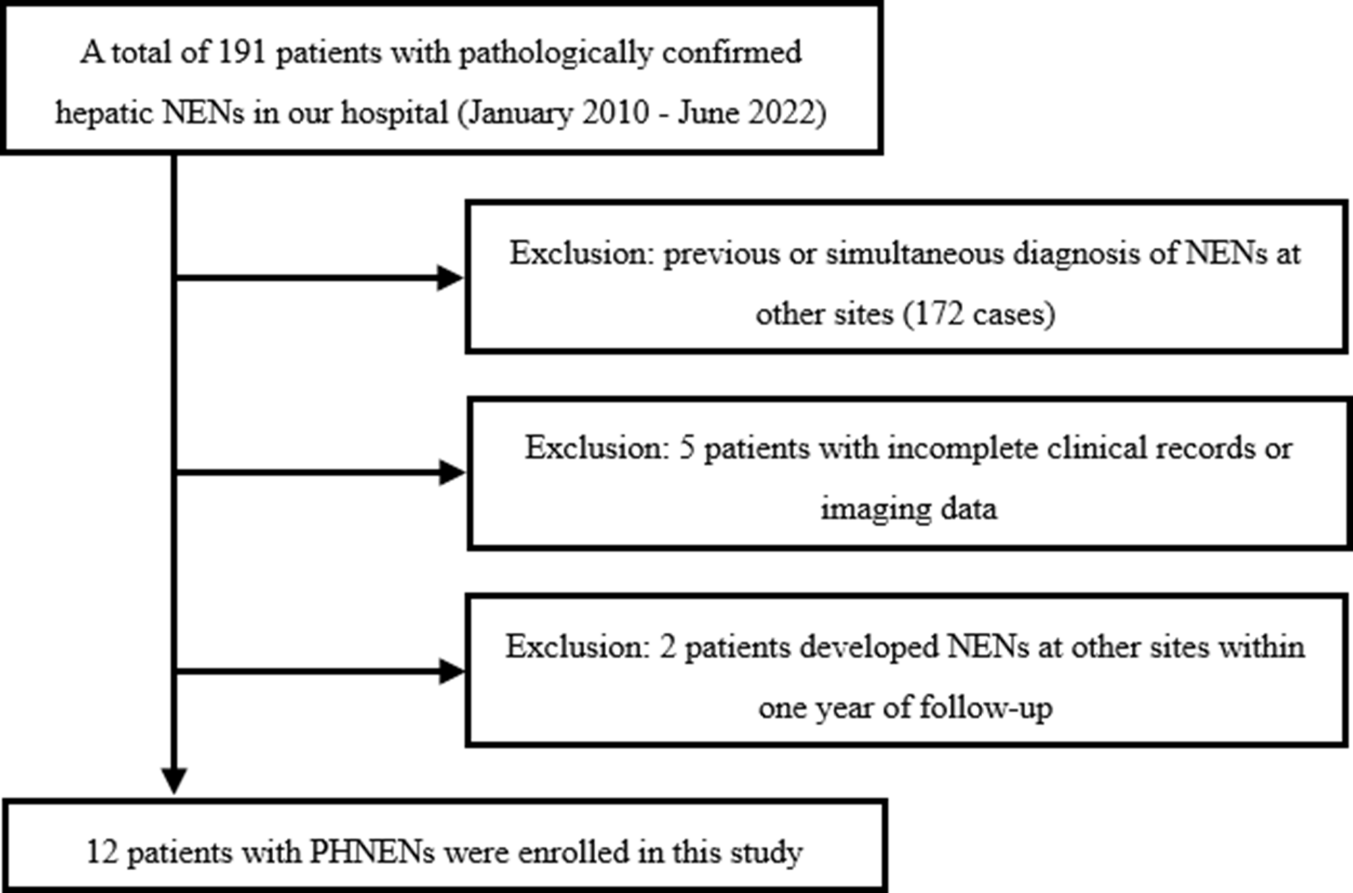
Flowchart depicting the process of case enrollment.

We conducted a retrospective search of our institution’s database to identify patients who were diagnosed with hepatic neuroendocrine neoplasms (NENs) between January 2010 and July 2022. The inclusion criteria encompassed the following aspects: (a) patients with histologically confirmed hepatic NENs by surgery or biopsy, (b) absence of NENs in other anatomical sites prior to diagnosis, at the time of diagnosis, and within one year after diagnosis, (c) availability of comprehensive histological descriptions in pathology reports, (d) availability of complete preoperative or biopsy imaging data including CT and MRI scans, and (e) availability of comprehensive clinical data. Exclusion criteria comprised: (a) incomplete clinical records, pathological data, or imaging data; and (b) presence of NENs in other anatomical sites before diagnosis, at the time of diagnosis, or within one year after diagnosis without excluding metastatic liver involvement. **Figure 1** illustrates the flow chart depicting patient selection.”

### 2.2 Instruments and methods

CT examinations were conducted using the SOMATOM Definition CT (Siemens), Ingenuity 128-slice CT (Philips), and Brilliance iCT 256-slice CT (Philips) systems. Volumetric scanning was employed for all cases. The scanning parameters included a tube voltage of 120kV, automatic tube current ranging from 179 to 250mAs, and a slice thickness and spacing of 5mm. A contrast agent (ultravist, concentration of 300mgI/ml) was administered via the cubital vein at a flow rate of 3.0-3.5 ml/s using a high-pressure syringe. Scanning during the arterial phase, venous portal venous phase, and delayed phase occurred at time intervals of 28s, 58s, and 120s after injection of the contrast agent.

The MR examination was conducted using Acheva 1.5T and Ingenia 3.0T superconducting MR scanners manufactured by Philips company. Fast spin-echo sequence was employed to acquire the images, including liver multi-directional T2-weighted imaging (T2WI), axial T1-weighted imaging (T1WI), axial T2 fat suppression sequence, dynamic contrast-enhanced (DCE)-MRI, enhanced axial T1WI, and coronal T1WI sequence. The parameters for T1WI were TR: 400-500 ms, TE: 10-15 ms; for T2WI were TR: 2000-2500 ms, TE: 60-70 ms; with a matrix size of 256×256 pixels. The slice thickness was set at 5 mm with an interslice gap of 1.0 mm. Gadolinium dimeglumine (Gd-DTPA) at a dose of 0.2 mmol/kg body weight was administered intravenously through the cubital vein using a high-pressure syringe at a flow rate of 2-3 ml/s.

### 2.3 Image analysis

Two experienced radiologists, with 8 and 12 years of expertise in abdominal imaging diagnosis respectively, independently reviewed the images of the enrolled cases in a blinded manner. The smallest observable lesion was defined as having a maximum diameter ≥5mm. The key observations include the following: lesion characteristics such as number, shape, boundary, and maximum diameter; pre-contrast and enhanced CT values at each phase; pre-contrast and enhanced CT values of normal hepatic parenchyma at each phase; T1WI signal and T2WI signal; presence of limited diffusion; relative enhancement compared to normal hepatic parenchyma at each phase; presence of cyst/necrosis and distribution pattern. Additionally, the presence or absence of blood, calcification, fat deposition, capsule formation, nonrim arterial phase hyper-enhancement, nonperipheral “washout”, halo enhancement, vascular/bile duct cancer embolus occurrence, intrahepatic bile duct dilation, local hepatic capsule collapse are also evaluated. The image observation indexes and standards primarily refer to the 2018 Liver Imaging Reporting and Data System (LI-RADS) (14). In case of disagreement, a final decision was made by another chief physician following an independent review.

### 2.4 Statistical analysis

Statistical analysis was performed using SPSS 23.0 software. The normality of the measurement data was assessed using the Kolmogorov-Smirnov test, and for those data that did not follow a normal distribution, standardization by Z score was conducted prior to conducting paired sample t-tests. A significance level of P < 0.05 was considered statistically significant.

## 3 Results

### 3.1 Clinical data

The study enrolled a total of 12 participants, consisting of 6 males and 6 females, with ages ranging from 40 to 89 years and a median age of 65.5 years (interquartile range, 23.25 years). Among them, one participant presented with recurrent diarrhea and another with right upper abdominal pain, while the remaining individuals were asymptomatic. None of the patients had a medical history of hepatitis or liver cirrhosis. Eight patients underwent contrast-enhanced CT examination, three patients underwent non-contrast CT examination (with one patient also undergoing contrast-enhanced MR examination), and one patient exclusively underwent contrast-enhanced MR examination. Histological specimens were obtained through biopsy in five cases and surgical procedures in seven cases. Hepatobiliary tumor markers including CEA, AFP, CA125, CA199, CA242, CA50 FER and SCCA were detected in all cases. Specifically, increased levels of AFP were observed in one case; elevated levels of CEA and CA199 were found in another case; increased levels of CEA, CA199, CA242, CA50 and FER were identified in yet another case; finally, a single case exhibited heightened SCCA levels. NSE was detected in three cases with significant elevation noted among two cases.

### 3.2 Imaging data

The initial radiological diagnosis of the 12 participants revealed hepatocellular carcinoma (n=7), liver metastases (n=3), liver adenoma (n=1), and intrahepatic cholangiocarcinoma (n=1); PHNENs were not considered in the diagnosis for any cases.

**Figure 2:**
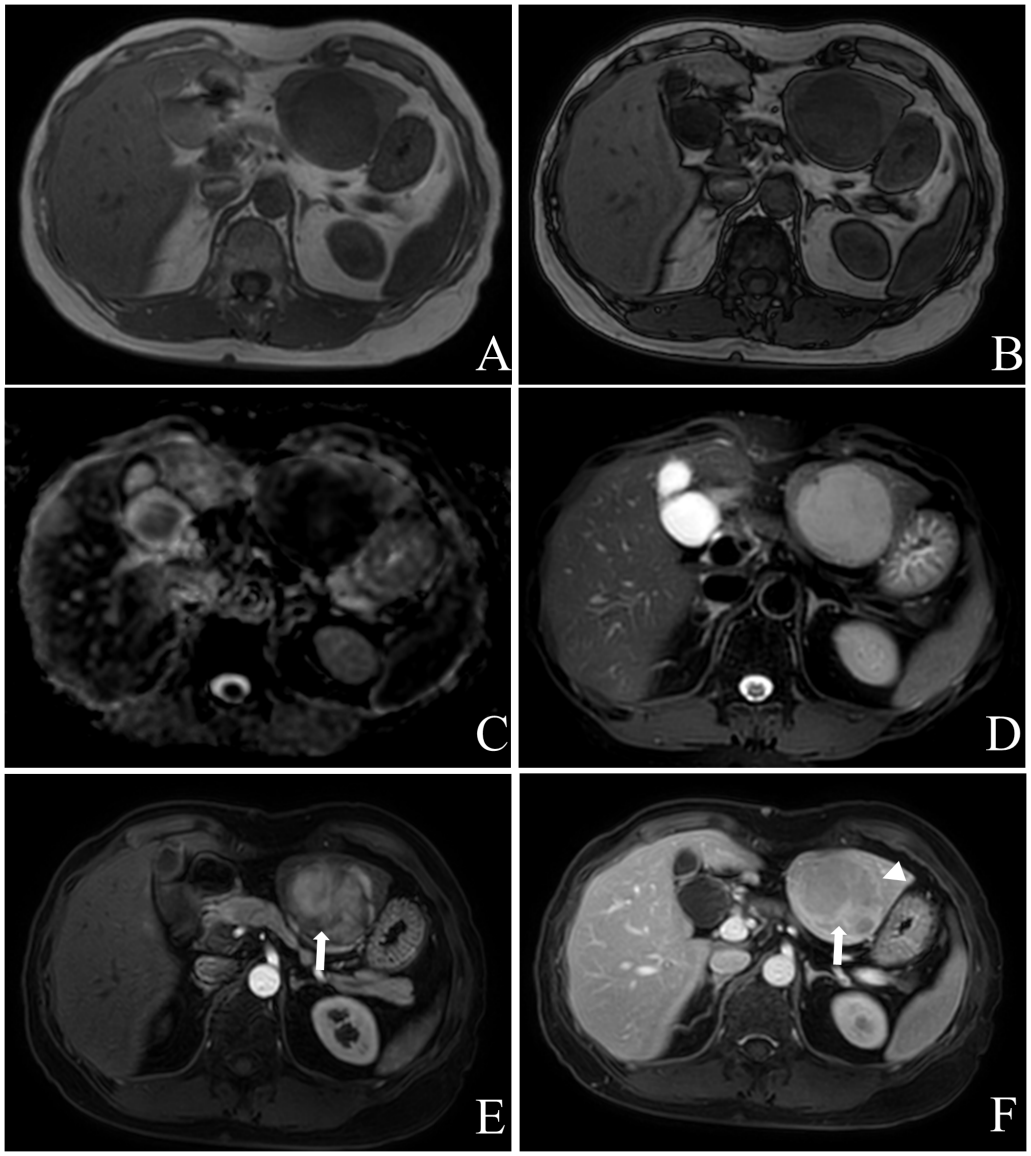
Female, aged 53 years, with an incidental liver mass and normal liver-related tumor markers; NSE was not tested. In November 2019, contrast-enhanced MR Imaging led to the preliminary diagnosis of hepatic adenoma. The liver exhibited three masses in S2, 3, and 8, characterized by low signal intensity on T1WI **(A)** and high signal intensity on T2WI **(D)**. These masses showed limited diffusion **(C)** without any apparent fat component **(A, B)**, and heterogeneous hyper-enhancement throughout the tumor on arterial phase with “wash-out” on venous portal and delayed phases, with localized inversion enhancement **(E, F)**, white arrow) and capsule **(F)**, white triangle) in the delayed phase. Subsequently, in December 2019, laparoscopic left hemihepatectomy and microwave ablation were performed for the treatment of the liver tumor. Postoperative pathology confirmed the presence of hepatic neuroendocrine tumor (G2). The patient remains alive with no evidence of extrahepatic NENs.

**Figure 3:**
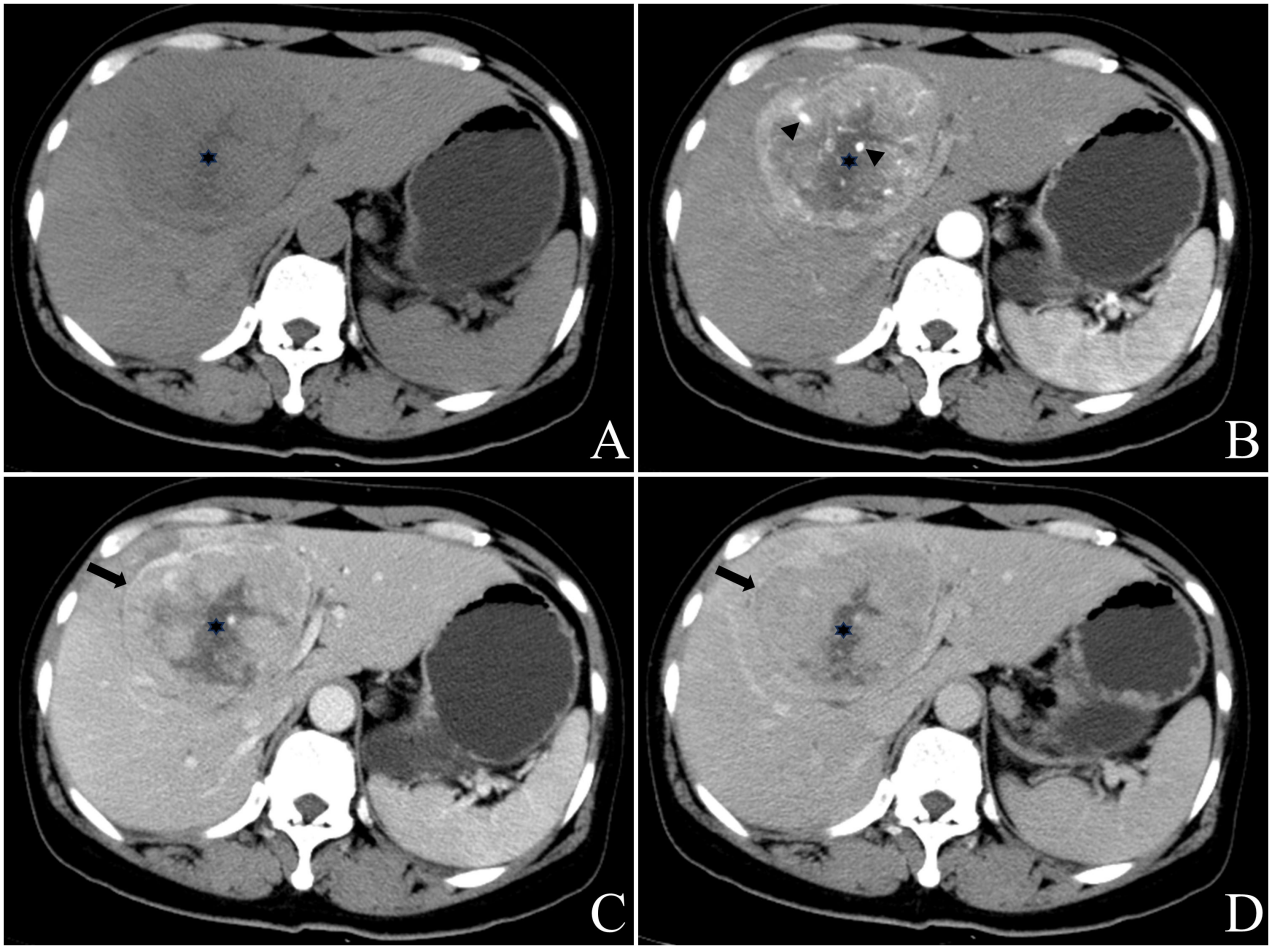
Female, aged 50 years, presented with right upper quadrant abdominal tightness and pain persisting for five days along with abdominal distention, occasional dizziness, and fatigue. Liver-related tumor markers as well as NSE levels were within normal range. A preliminary diagnosis of hepatocellular carcinoma with multiple metastases was made based on contrast-enhanced CT findings in May 2018. The masses located at hepatic S4 and 8 displayed a round mildly lobulated mass measuring up to a maximum cross-section size of approximately 90.4mm x80.5mm; it had well-defined boundaries and appeared slightly heterogeneous hypodense on pre-contrast CT images. Irregular circumferential hyperenhancement was observed on arterial phase imaging along with thickened and thickened tumor blood vessels **(B)**, black arrow head), while peripheral “washout” on venous portal phase and delayed phase accompanied by incomplete capsule **(C, D)**, black arrow). A radially low attenuation area with no apparent enhancement is seen in the center of the mass **(A-D)**, black asterisk). CT value of the most obvious enhanced area/normal hepatic parenchyma: pre-contrast 46.3/52.1; arterial phase 95.0/62.2; venous portal phase 92.9/101.8; delay phase 78.4/87.6. Enlarged right half liver resection was performed in May 2018. Postoperative pathology: neuroendocrine carcinoma (G3). EP chemotherapy regimen was performed after surgery, and the patient remains alive with no evidence of extrahepatic NENs.

**Figure 4:**
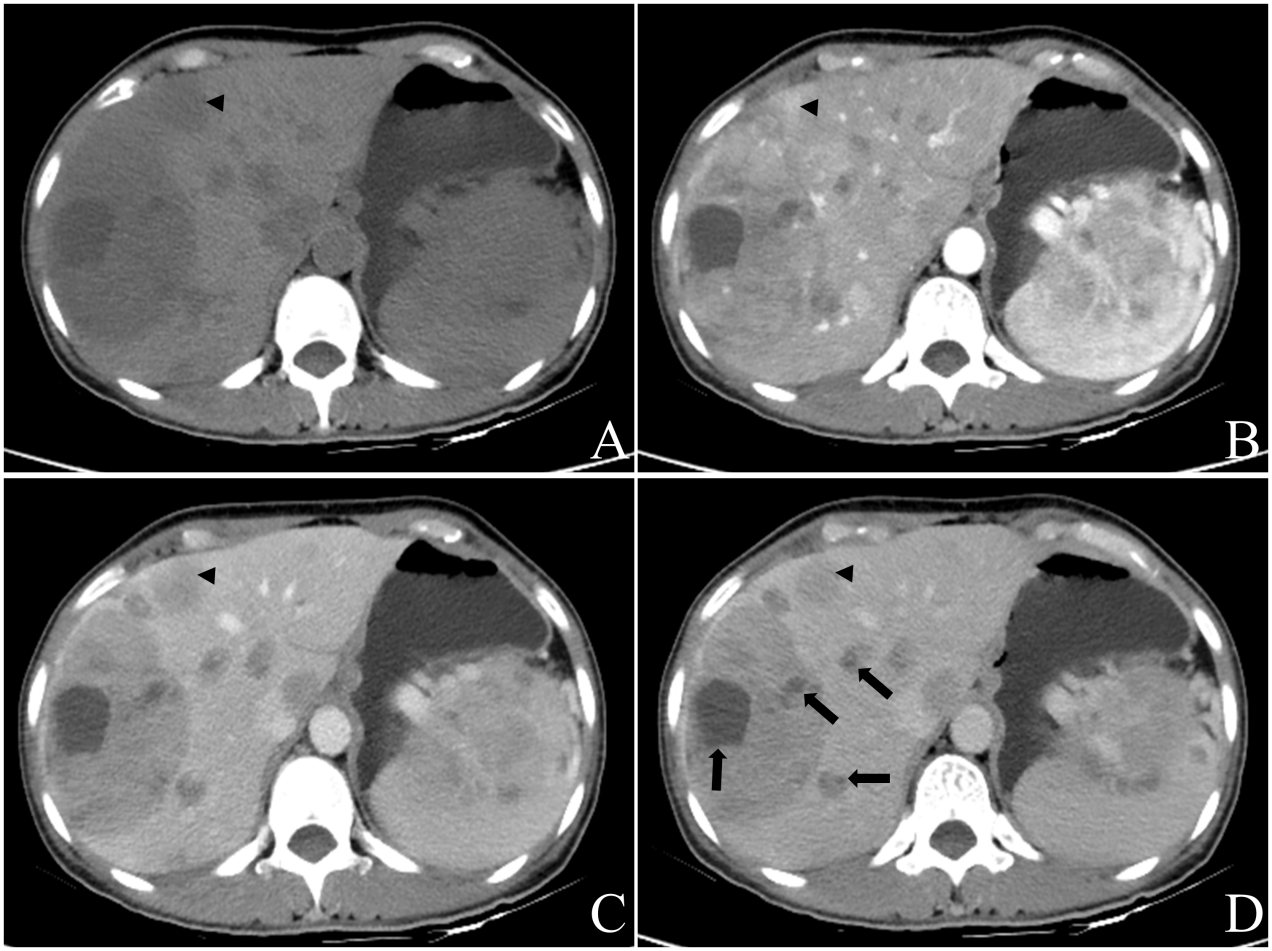
Female, aged 49 years, incidentally discovered multiple liver masses. The levels of CEA were measured at 10.53ng/ml, PIVKA at 56mAU/ml, CA199 at 237.15U/ml, and NSE levels were within the normal range. Based on contrast-enhanced CT in July 2022, the preliminary diagnosis included: (1) gastric malignant stromal tumor with multiple hepatic metastases; (2) possibility of primary liver tumors not excluded due to the presence of a large mass in the right lobe. Multiple round-like masses were observed throughout the liver, with the largest lobulated mass measuring approximately 102.7mm x 71.9mm in cross-section located in S7 and S8 segments. The contrast-enhanced scan revealed irregular annular arterial phase hyper-enhancement and peripheral “washout” in venous portal phase and delayed phase. Some smaller lesions exhibited whole-tumor enhancement **(A-D)**, black triangle). Additionally, multiple cystic foci were identified mainly in the periphery of the lesion as well as smaller lesion **(D)**, black arrow). Biopsy results confirmed a neuroendocrine tumor (G2) for this liver mass, and the patient remains alive with no evidence of extrahepatic NENs. The pathology report for another operation involving caudal pancreatic space occupying lesion showed solid pseudopapillary tumor of the pancreas.

A total of 82 masses were identified, with 3 patients having a single mass and 9 patients having multiple masses (n≥2). The maximum diameter of the masses ranged from 25.7mm to 116.2mm. Most of the masses exhibited a round or round-like shape, while some showed shallow lobulation. Noncontrast CT scans demonstrated hypodensity, low signal intensity on T1WI, high signal intensity on T2WI, high signal intensity on DWI, and low signal intensity on ADC imaging (**Figure 2**). Contrast-enhanced CT scans (6/8) and contrast-enhanced MR scans (1/2) displayed similar rim arterial phase hyper-enhancement, followed by decreased density/signal intensity in the venous portal phase and delayed phase respectively, primarily exhibiting peripheral washout patterns (**Figures 3**, **4**). Mild and progressive enhancement was observed in two cases in contrast-enhanced CT. One case exhibited heterogeneous hyper-enhancement throughout the tumor on arterial phase with decreased enhancement on venous portal and delayed phases as well as delayed enhancement was found in localized areas of the tumor, and it showed inversion enhancement (**Figure 2**). Three cases presented an incomplete capsule structure (**Figure 3**) while one case had a complete capsule formation surrounding it. One case displayed nonrim arterial phase hyper-enhancement and nonperipheral “washout”. Halo enhancement was detected in eight cases. Cyst/necrosis was observed in seven out of all enhanced cases; among them five cases predominantly exhibited peripheral distribution (**Figure 4**), one case predominantly showed central distribution (**Figure 3**), and one case had scattered distribution. All lesions lacked fat content (**Figure 2**), calcification, hemorrhage, vessel or bile duct tumor thrombus, lymph node metastasis, intrahepatic bile duct dilatation, or local collapse of the liver capsule. There were no knot-in-knot sign or Mosaic structures sign. The imaging findings of the 12 cases are summarized in **Table 1**.

**Table 1:** Summary of CT and MR findings of 12 cases.

SN	Gender	Age	Imaging method	Quantity	Location	Form	Boundary	Maximum lesion cross section (mm)	T1WI signal	T2WI signal	Diffusion-limited	CECT/CEMR	Cyst/necrosis	Capsule	Nonrim arterial phase hyperenhancement	Nonperipheral washout	Halo enhancement	Others
1	Male	40	CECT	1	V, VI, VII	Round	Clear	65.9×58.7	NA	NA	NA	Hypo-enhancement than normal liver	Mainly peripheral, few in the center	Negative	Negative	Negative	Negative	Negative
2	Female	41	CECT	17	Whole liver, except SI	Most round, few round-like with shallow lobulation	Clear	96.8×64.8	NA	NA	NA	Similar rim arterial phase hyper-enhancement and then decreased to low attenuation	Mainly peripheral, few in the center	Negative	Negative	Negative	Positive, with increased perfusion of the surrounding liver parenchyma	Negative
3	Male	68	CECT	1	VIII	Round	Clear	37.3×32.1	NA	NA	NA	Similar rim arterial phase hyper-enhancement and then decreased to low attenuation	Negative	Negative	Negative	Negative	Positive	Negative
4	Female	50	CECT	9	IV, VIII	Round-like with shallow lobulation	Clear	90.4×80.5	NA	NA	NA	Similar rim arterial phase hyper-enhancement and then decreased to low attenuation	Radial necrosis in the center	Incomplete	Negative	Negative	Positive	Negative
5	Female	63	CECT	2	VI, VII, VIII	Round-like with shallow lobulation	Clear	116.2×160.1	NA	NA	NA	Hypo-enhancement than normal liver	Radial necrosis in the center and peripheral cyst/necrosis	Negative	Negative	Negative	Negative	Negative
6	Female	49	CECT	33	Whole liver	Round with shallow lobulation	Clear	102.7×71.9	NA	NA	NA	Similar rim arterial phase hyper-enhancement and then decreased to low attenuation	Mainly peripheral, few in the center	Negative	Negative	Negative	Positive	Negative
7	Male	71	CECT	3	IV, V, VI, VIII	Round-like with shallow lobulation	Clear	93.9×83.0	NA	NA	NA	Similar rim arterial phase hyper-enhancement and then decreased to low attenuation	Peripheral and central cyst/necrosis	Incomplete	Negative	Negative	Positive	Negative
8	Male	70	CECT	5	IV, V, VI	Round-like with shallow lobulation	Clear	34.7×27.8	NA	NA	NA	Similar rim arterial phase hyper-enhancement and then decreased to low attenuation	Peripheral and central cyst/necrosis	Incomplete	Negative	Negative	Positive	Negative
9	Female	73	Non-CECT	5	II, IV, VI, VII, VIII	Round	Clear	44.9×38.7	NA	NA	NA	NA	Negative	Negative	Negative	Negative	Negative	Negative
10	Male	78	Non-CECT	2	VIII	Round	Clear	38.7×29.9	NA	NA	NA	NA	Negative	Negative	Negative	Negative	Negative	Negative
11	Female	52	Non-CECT and CEMR	3	II, III, VIII	Round	Clear	51.8×45.1	Slightly lower signal intensity	Slightly higher signal intensity	Positive	Hyperenhancement in arterial phase followed by attenuation to slightly hypointensity	Negative	Whole	Positive	Positive	Positive	Negative
12	Male	89	CEMR	1	VIII	Round-like with shallow lobulation	Less clear	25.7×17.5	Slightly lower signal intensity	Slightly higher signal intensity	Positive	Similar rim arterial phase hyper-enhancement and then decreased to isointensity	Negative	Negative	Negative	Negative	Positive	Negative

1, CECT, Contrast-enhanced CT; 2, CEMR, contrast-enhanced MR; 3, NA, Not applicable; 4, Others include fat, calcification, hemorrhage, vessel or bile duct tumor thrombus, lymph node metastasis, intrahepatic bile duct dilatation, local collapse of the liver capsule, knot-in-knot sign and mosaic structures sign.

**Figure 5:**
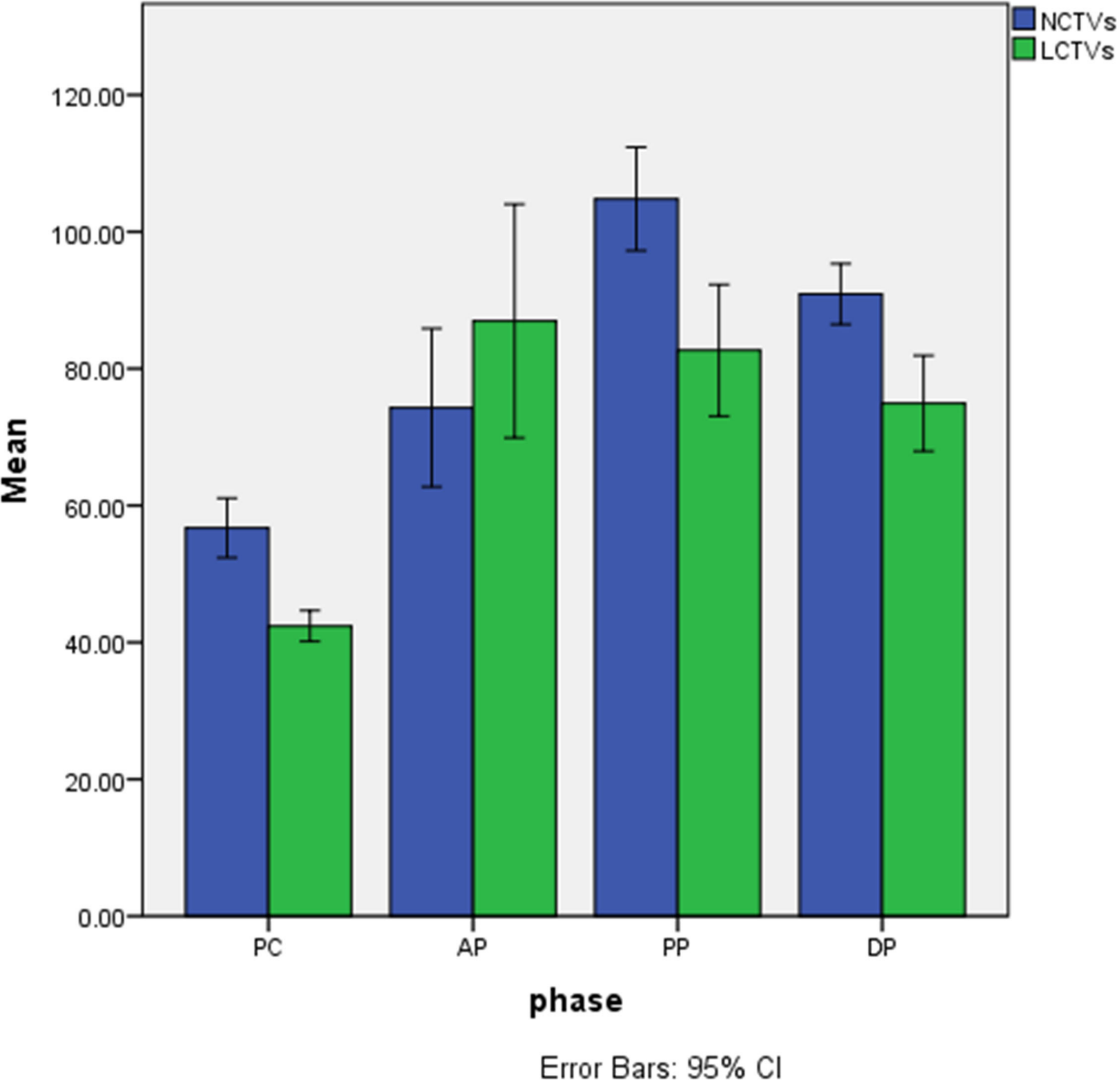
Histogram of CT values of lesions and normal hepatic parenchyma on each phase. 1、NCTVs, CT values of normal hepatic parenchyma; 2、LCTVs, CT values of lesions; 3、PC, pre-contrast; AP, arterial phase; PP, venous portal phase; DP, delayed phase.

In 8 patients performed contrast-enhanced CT, we selected the largest mass and the area with the most obvious enhancement as well as normal hepatic parenchyma for measurement, recorded the CT values of the mass and normal hepatic parenchyma on pre-contrast, arterial phase, venous portal phase and delayed phase. Measurements of FOV area ≥15mm^2^ are required, and blood vessels, calcification, cyst/necrosis et al. should be avoided during measurement. The results are shown in **Table 2** and **Figure 5**.

**Table 2:** CT values of lesions and normal hepatic parenchyma on each phase.

ID	CT values (HU)							
	lesions				normal hepatic parenchyma			
	PC	AP	PP	DP	PC	AP	PP	DP
1	44.1	58.6	77.0	77.4	61.4	67.0	97.2	86.3
2	38.4	107.6	91.9	86.3	47.9	89.3	100.5	92.2
3	44.4	94.6	74.8	66.1	58.7	67.7	98.3	87.9
4	46.3	95.0	92.9	78.4	52.1	62.2	101.8	87.6
5	39.7	53.9	65.4	67.3	64.5	87.9	110.2	98.2
6	41.7	101.2	91.8	82.0	58.1	94.2	109.5	95.4
7	43.9	82.2	72.5	63.1	56.1	67.1	97.6	83.6
8	40.6	102.7	95.1	78.8	55.0	59.0	123.3	96.1

1、PC, pre-contrast; 2、AP, arterial phase; 3、PP, venous portal phase; 4、DP, delayed phase.

### 3.3 Results of statistical analysis

**Figure 6:**
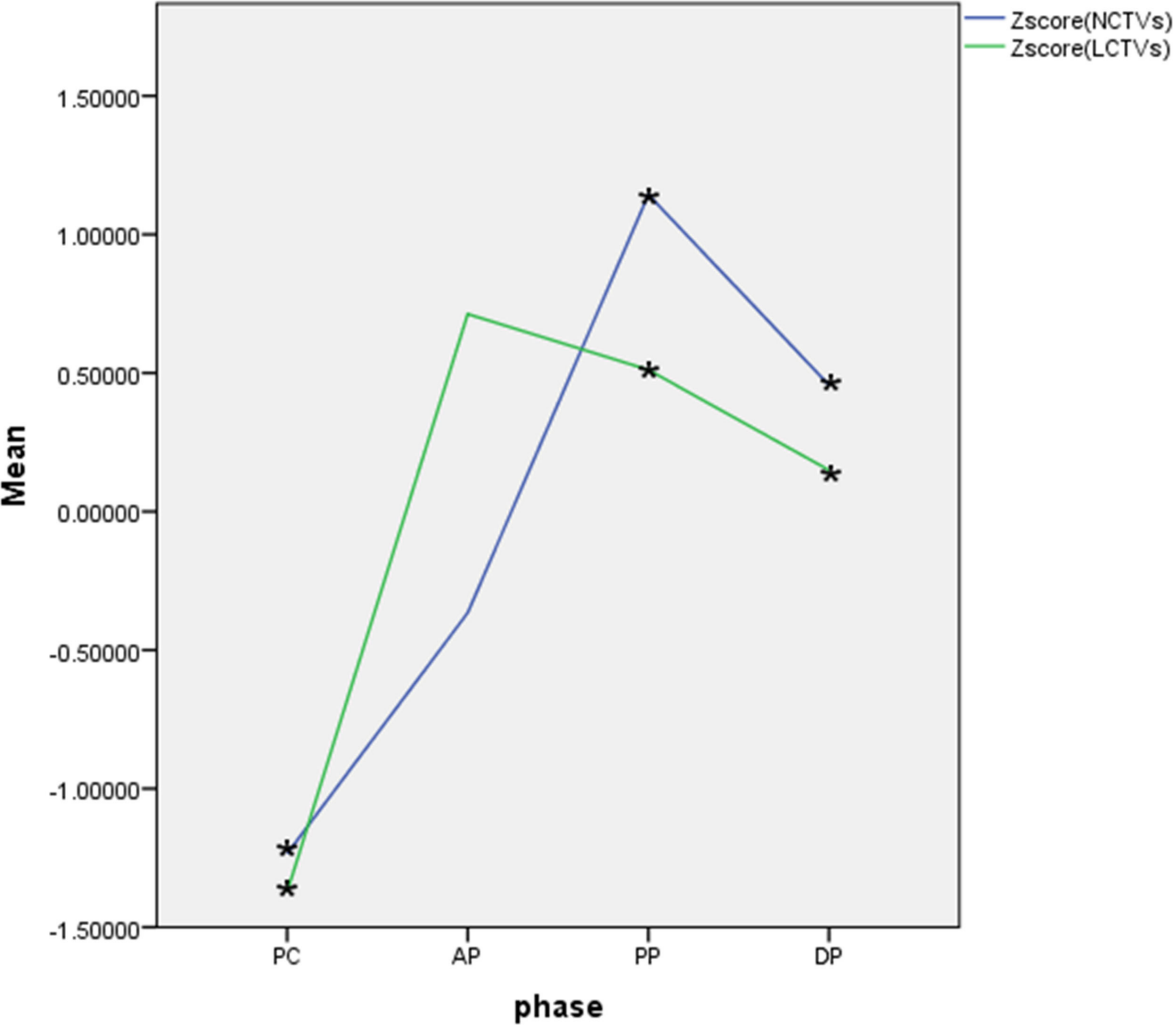
Curve of Z-score CT values of lesions and normal hepatic parenchyma on each phase. 1、Zscore(NCTVs), Z-score of CT values of normal hepatic parenchyma; 2、Zscore(LCTVs), Z-score of CT values of lesions; 3、PC, pre-contrast;AP, arterial phase; PP, venous portal phase; DP, delayed phase; 4、·*indicates that the difference was statistically significant.

The normality of CT values for masses and normal hepatic parenchyma in each phase of contrast-enhanced CT was assessed using the Kolgomorov Smirnov test, which revealed a deviation from the normal distribution. Z-score standardization was applied to address this data, and a graph was plotted followed by paired sample t-test analysis. The masses exhibited an outflow type enhancement pattern, with statistically significant differences observed in CT values between the masses and normal liver parenchyma during pre-contrast, venous portal phase, and delayed phase (P < 0.05). However, no statistically significant difference was found in CT values during the arterial phase (P=0.179), as depicted in **Figure 6**.

## 4 Discussion

Primary hepatic neuroendocrine neoplasms (PHNENs) are very rare, accounting for about 0.4% of all NENs cases (3). Most cases of PHNENs occured in Asian people (15). Most patients are asymptomatic, a few may have upper abdominal pain, and about 5% of patients may have carcinoid syndrome. Tumor markers such as AFP, CEA, CA1-99, PIVKA-II, and CA7-24 are more normal, and their negative results help distinguish them from other tumors in the liver. However, chromoprotein granules (CgA), neuron-specific enolase (NSE) and 24h-5 hydroxy-indole acetic acid (5-HIAA) are effective methods for the diagnosis of NENs, which are related to tumor size and stage, and NSE and CgA can even predict postoperative recurrence. The exact origin of PHNENs is unclear, but it is generally thought to originate from neuroendocrine cells in the epithelium of the hepatobiliary tract, ectopic adrenal or pancreatic tissue, or liver stem cells (16, 17). It is difficult to distinguish primary hepatic NENs from metastatic hepatic NENs based on pathological evidence alone. Therefore, clinical information is essential for the diagnosis of primary or metastatic hepatic NENs. Preoperative thorough examination, intraoperative examination, and postoperative follow-up are very important to determine whether the lesions originate in the liver (18), but there is no definitive standard for the duration of follow-up required for the diagnosis of PHNENs. In this study, all the enrolled patients were confirmed to be PHNENs as PHNENs by histopathology, preoperative systemic evaluation and one-year postoperative follow-up.

The imaging features of PHNENs have been summarized in previous literature (19–25) as follows: (1) Tumors can occur as single or multiple lesions with no significant difference in their distribution between the left and right lobes of the liver; (2) Heterogeneity is observed in tumors, presenting as uneven low attenuation on pre-contrast CT and mixed long T1 and long T2 signals on MRI; (3) Significant enhancement of tumor parenchyma is observed on the arterial phase, while a decrease in enhancement is noted on the venous portal phase and delayed phase; (4) Calcification and fat deposition within the lesion are rare occurrences, as well as cancer thrombus formation within blood vessels or bile ducts.

The imaging characteristics of this cohort are in substantial agreement with previous literature reports. Upon reviewing the imaging data of these patients, referencing the 2018 Liver Imaging Reporting and Data System (LI-RADS), several noteworthy imaging findings were identified by the author. (1) Compared to hepatocellular carcinoma, PHNENs often exhibit similar rim arterial phase hyper-enhancement and peripheral “washout” on venous portal phase and delayed phase. Approximately 77.7% (7/9) of all contrast-enhanced cases in this group demonstrated this enhancement pattern. Previous studies (26) have reported that PHNENs tend to show intense reinforcement on the arterial phase due to their abundant arterial blood supply. The different enhancement patterns observed in the venous portal vein phase and delayed phase are believed to be associated with a balance between vasoactive substances and fiber activities secreted by NENs. Furthermore, this balance may vary with tumor size, as lesions exhibiting continuous strengthening were found to contain more fibrous components. Most patients in this group showed decreased enhancement during the venous portal phase and delayed phase, which is thought to be related to a higher concentration of vasoactive substances within the lesions. In one case, early high enhancement followed by subsequent reduction was observed throughout most areas of mass in the contrast-enhanced MR, while some regions exhibited progressive enhancement; these findings were attributed to a higher content of local fiber components within the tumor. (2) There was a high likelihood of cyst/necrosis, accounting for 77.7% (7/9) of all contrast-enhanced cases in this group, even some small lesions would appear, with exhibiting a tendency towards peripheral distribution (5/7). In fact, the distinction between cystic degeneration and necrosis based solely on CT or MR findings poses a challenging task. Hence, both are collectively analyzed and counted in this study. Previous observations have indicated that tumor necrosis often arises due to inadequate blood supply within rapidly growing tumors, primarily occurring at the tumor center. The presence of surrounding cystic/necrotic areas observed in this study remains unexplained by the current theory. Based on the pathological findings from a previous investigation on pancreatic NENs (27), it is postulated that internal hemorrhage/necrosis within tumors contributes to the formation of cystic foci as visualized through imaging techniques. Certainly, two patients in this cohort exhibited a centrally distributed area of low attenuation, potentially attributed to tumor necrosis. Previous literatures (7, 23) have all described the solid-cystic characteristics of PHNENs but did not provide further elucidation on the distribution characteristics of cystic changes or necrosis. Li et al. (19) reported 13 cases of PHNENs, among which five cases presented central low-density regions and suggested that the astral sign within the lesion’s center was one characteristic. This perspective contradicts the findings of this study. The reason for this contradiction may stem from the rarity of PHNENs and the limited size of the study sample, necessitating further case observations to address this issue.

The initial imaging diagnosis of the 12 patients in this cohort comprised hepatocellular carcinoma (n=7), hepatic metastatic tumor (n=3), hepatic adenoma (n=1), and intrahepatic cholangiocarcinoma (n=1). An analysis of the factors contributing to misdiagnosis is presented. (1) Hepatocellular carcinoma: In this study, the majority of cases were misdiagnosed as hepatocellular carcinoma. The reasons for misdiagnosis were analyzed as follows. The arterial phase of PHNENs exhibited high enhancement followed by clearance in the venous portal and delayed phases, resembling hepatocellular carcinoma. However, unlike typical hepatocellular carcinoma, the high enhancement and clearance were distributed around the lesion in a ring-like shape. Additionally, some cases displayed cystic/necrotic features with a tendency to distribute around the lesion rather than exhibiting central necrosis commonly seen in hepatocellular carcinoma. All misdiagnosed cases had no history of hepatitis or cirrhosis, and liver tumor markers such as AFP were not elevated. Furthermore, even when the mass was large, there was no presence of venous portal tumor thrombus. Nevertheless, there is a considerable overlap in clinical history, laboratory examination, and imaging findings between PHNENs and hepatocellular carcinoma (28, 29). (2) Liver metastases: Three cases were misdiagnosed as metastatic tumors, two of which had a history of esophageal cancer surgery. The other case was also found to have solid pseudopapilloma of the pancreas (misdiagnosed as gastric stromal tumor at that time), which posed challenges in accurate diagnosis. Most liver metastases exhibit certain characteristics similar to primary tumors. In cases of liver metastases from esophageal cancer and gastrointestinal stromal tumor, circular and moderate enhancement is commonly observed, while arterial phase high enhancement is rarely seen. (3) Hepatic adenoma: Imaging findings of different types of hepatic adenomas exhibit significant variations. The typical features of this condition include pre-contrast CT findings with slightly decreased attenuation, variable MR T1WI signal intensity, susceptibility to bleeding or steatosis, short and relatively homogeneous arterial phase enhancement on contrast-enhanced scans, as well as isodensity/signal characteristics in the venous portal and delayed phases. In this group, one case was misdiagnosed as hepatic adenoma primarily due to the presence of an envelope-like appearance and slight wash-out in the venous portal and delayed phases, leading to misdiagnosis. (4) Cholangiocarcinoma: The majority of manifestations are accompanied by both intra and extralesional bile duct dilation, as well as elevated CEA levels. Typically, cholangiocarcinomas exhibit continuous and progressive enhancement following contrast administration. However, the extent of arterial enhancement is often less conspicuous compared to that of PHNENs. In this particular group, one case was initially misdiagnosed as cholangiocarcinoma due to slightly higher contrast enhancement on arterial phase compared to normal hepatic parenchyma, and slightly wash-out. Nevertheless, the observed enhancement pattern differed slightly from that typically seen in cholangiocarcinomas, with no evidence of intrahepatic bile duct dilatation or abnormal tumor markers. (5) Metastatic hepatic neuroendocrine neoplasms (MPHNENs): The differential diagnosis of PHNENs and MHNENs is a complex challenge difficult for both pathologists and radiologists. Some radiologists have conducted exploratory studies on the MR manifestations of both tumor types and propose that certain features such as large, solitary or rapidly growing nodules with lobulated or irregular contours, capsule-like enhancement, heterogeneous signals, or lower apparent diffusion coefficient (ADC) values may potentially support the diagnosis of PHNENs compared to metastatic ones (25). The presence of multiple lesions, tumor size less than 6.3 cm, and a hepatocellular carcinoma-like enhancement pattern have been identified as significant independent factors for differentiating secondary from PHNENs (30). In this study group, six cases (6/12) had a maximum diameter less than 6.3 cm deviating from previous literature findings. Therefore, further case studies are recommended to enhance the imaging-based differential diagnosis between these two entities.

In summary, PHNENs exhibit distinct clinical and imaging characteristics. In patients without clinical symptoms or a history of liver disease, with normal liver tumor markers levels such as AFP but significantly elevated NSE levels, the presence of multiple intrahepatic masses with peripheral cyst/necrosis and similar rim arterial phase hyper-enhancement along with peripheral ‘washout’ in venous portal and delayed phases on CT or MR imaging should raise suspicion for hepatic NENs. The diagnosis of primary or metastatic disease should be made in conjunction with comprehensive systemic evaluation and long-term follow-up.

Although this study presents novel findings regarding the imaging characteristics of PHNENs, there are still certain limitations that need to be addressed. Firstly, the utilization of different imaging devices for data collection hampers results comparability. Secondly, due to the rarity of PHNENs, a limited number of cases were included in this study. Lastly, while no evidence of extra-hepatic NENs was observed during the 1-year follow-up period, it is important to acknowledge that the possibility of their occurrence after a longer duration cannot be completely ruled out.

## Data availability statement

The raw data supporting the conclusions of this article will be made available by the authors, without undue reservation.

## Ethics statement

The studies involving humans were approved by Clinical Research Ethics Committee of the First Affiliated Hospital of Xiamen University. The studies were conducted in accordance with the local legislation and institutional requirements. The ethics committee/institutional review board waived the requirement of written informed consent for participation from the participants or the participants’ legal guardians/next of kin because In this study, the imaging, pathological and medical records generated during the previous diagnosis and treatment of patients were retrospectively used. The exemption from informed consent will not adversely affect the rights and health of the subject. Patients were numbered during the study, and personal information such as name and image number was blocked. Written informed consent was not obtained from the individual(s) for the publication of any potentially identifiable images or data included in this article because The Clinical Research Ethics Committee of the First Affiliated Hospital of Xiamen University approved the informed consent exemption for this study.

## Author contributions

XY: Writing – original draft, Writing – review & editing. YL: Conceptualization, Data curation, Formal analysis, Writing – original draft, Writing – review & editing. ZL: Data curation, Investigation, Writing – original draft, Writing – review & editing. XC: Formal analysis, Visualization, Writing – review & editing.
